# Application of metal-based nanoparticles in targeted drug delivery to breast cancer stem cells

**DOI:** 10.1186/s11671-026-04772-7

**Published:** 2026-07-04

**Authors:** Zahra Gholizadeh, Saghar Yousefnia

**Affiliations:** 1 Department of Physics, Semnan University, Semnan, Iran; 2 Department of Cell & Molecular Biology, Semnan University, Semnan, Iran

**Keywords:** Breast cancer, Breast cancer stem cells (BCSCs), Metal nanoparticles (MNPs), Photothermal therapy, Gold/Copper/Iron oxide/Silver nanoparticles, Targeted drug delivery

## Abstract

Breast cancer stem cells (BCSCs) are a minor population within breast cancer tissue, causing cancer development and cancer recurrence due to stem-like and malignant properties, including self-renewal, proliferation, differentiation, migration, invasion, metastasis, and resistance to chemo/radio therapy. Treatment of these types of cancer cells requires targeted approaches to address the unique properties of BCSCs. Metal nanoparticles (MNPs) can be applied as drug delivery nanosystems in the treatment of BCSCs. Breast cancer treatments by MNP-based targeted drug delivery are essential because they reduce patient discomfort and recovery time, while enabling more precise and targeted therapy, potentially reducing off-target effects when appropriately designed and dosed. The size, shape, surface characteristics, and unique properties of MNPs, including their optical and physicochemical characteristics, safety profiles that are highly dependent on particle design and administration parameters, stability, circulation half-life, biodistribution, and high drug release efficiency, make them highly reactive and versatile for a variety of biomedical applications. However, their safety profiles are strongly influenced by particle design, dosage, and administration parameters, as high concentrations may induce dose-dependent toxicity, including ROS-mediated cellular damage. This review focuses on applications of various MNPs, including gold, copper, iron oxide, and silver, their surface modifications, and combination with photothermal therapy and several therapeutic agents in the drug delivery of BCSCs. This review also highlights the advantages, current challenges, and prospects of MNPs in drug delivery systems. These promising approaches have significant potential for developing new strategies for invasive treatment of breast cancer.

## Introduction

Breast cancer is the most common cancer in women around the world. While breast cancer mortality rates have significantly decreased, it remains a leading cause of cancer death among women [1]. In 2024, according to the American Cancer Society, 310,720 new cases of invasive breast cancer were diagnosed, and 42,250 people died from breast cancer in the US [2]. Breast cancer is classified into ductal and lobular types, each of which may be in situ (non-invasive) or invasive disease, with multiple subtypes defined by histopathological features. In addition, breast cancer is categorized into molecular subtypes based on the expression of estrogen receptor (ER), progesterone receptor (PR), human epidermal growth factor receptor 2 (HER2), and Ki-67. These molecular subtypes include luminal A and luminal B (ER+/PR+/HER2 − or ER+/PR−/HER2−), basal-like or triple-negative breast cancer (ER−/PR−/HER2−), HER2^+^, and claudin-low tumors [3]. The main causes of breast cancer are categorized into lifestyle factors, including overweight, alcohol consumption, diet, and genetic factors, such as family history, and mutations in *BRCA1* and *BRCA2* [1]. In addition, epigenetic factors, alterations in DNA methylation, histone modification patterns, and miRNAs expression, can affect gene expression, oncogenes and tumor suppressor genes and contribute to breast cancer development [1, 4].

There are some common and traditional therapies for breast cancer, such as surgery, chemo/radiotherapy, and hormone therapy [1]. Although these therapies can be effective in a subset of patients, there are many side effects, and most of the time, recurrence is the main difficulty in breast cancer therapy [5]. The cellular diversity within tumors presents a significant challenge for effective cancer treatment [6]. Tumors are composed of various cell types, including cancer stem cells (CSCs) and more differentiated cancer cells. CSCs possess several characteristics that contribute to the complexity of cancer [7–9]. Genetic and epigenetic alterations lead to the dysregulation of multiple signaling pathways, including MAPK, PI3K/Akt/NF-κB, TGF-β, Hedgehog, Notch, Wnt/β-catenin, and Hippo, promoting the transformation of normal stem cells, progenitor cells, or differentiated cells into CSCs. In breast cancer, BCSCs represent a small population of the tumor; however, they play a critical role in treatment resistance, tumor recurrence, and metastasis [10]. Conventional therapies for breast cancer are limited by systemic toxicity, poor specificity, and failure to eliminate breast cancer stem cells, leading to recurrence and metastasis. These challenges highlight the need for advanced treatment strategies, as nanotechnology, particularly nanoparticle-based drug delivery systems, offers targeted delivery, improved drug accumulation, and the potential to overcome drug resistance. BCSCs require targeted approaches to address the unique properties of CSCs to achieve successful treatment outcomes [9, 11]. BCSCs express various specific markers, including CD44+/CD24-, CD326 (EpCAM), epithelial specific antigen (ESA), and aldehyde dehydrogenase (ALDH). These specific biomarkers provide an opportunity for designing smart nanosystems that are conjugated with ligands and peptides, targeting BCSCs, specifically [12]. In recent years, there has been a significant increase in research focusing on personalized medicine approaches for treating diseases. This involves treatments, including immunotherapy and targeted therapy to target the specific molecules of individual patients [13–15]. Furthermore, researchers are exploring the various nanomaterials as drug delivery systems. These include nanopolymers like polyethylenimine, PLGA, chitosans, collagen, and gelatin, as well as phytochemical-based nanoparticles and inorganic and metal materials such as gold, silver, iron oxide, and copper [16–19]. Metal nanoparticles (MNPs) have attracted considerable attention in biomedical applications due to their unique magnetic responsiveness, biocompatibility, and tunable surface chemistry. These nanoparticles are widely utilized as contrast agents in magnetic resonance imaging (MRI), enabling enhanced diagnostic sensitivity and noninvasive disease monitoring [20]. MNPs offer tunable surface functionalization, enhanced cellular uptake, and controlled drug release. These advantages make them promising carriers for the targeted therapy of cancer [21]. Traditional therapies such as chemotherapy, radiotherapy, and targeted agents and monoclonal antibodies often fail to fully eradicate BCSCs due to their intrinsic resistance mechanisms and their ability to evade immune surveillance [10]. In contrast, MNPs can improve therapeutic efficacy through multiple mechanisms, including enhanced drug delivery via the enhanced permeability and retention (EPR) effect, surface functionalization for active targeting of BCSC-specific markers, and the ability to act as therapeutic agents themselves (e.g., through photothermal or radiosensitizing effects). Additionally, MNPs may enable controlled and sustained drug release, potentially overcoming resistance associated with conventional dosing [21].

The goal of this review is to explore the development of more precise and effective drug delivery systems capable of selectively targeting BCSCs. It focuses on the applications of various MNPs and their surface modifications in BCSC-targeted drug delivery. In particular, the advantages, current challenges, and prospects of metal-based nanomaterials, such as gold, silver, iron oxide, and copper, are discussed in the context of their therapeutic applicability. In this review, nanoparticles are defined as materials possessing at least one dimension within the 1–100 nm size range, in accordance with standard nanotechnology definitions. Collectively, these approaches offer promising opportunities for the development of novel strategies to combat invasive breast cancer.

## Methodology

A literature search was conducted to identify relevant articles on BCSCs, their biomarkers, and mechanistic pathways of malignancy, metal nanoparticles and their synthesis, as well as their application in cancer drug delivery targeting BCSCs. The search was performed across three major scientific databases: PubMed, ScienceDirect, and Google Scholar. The search was limited to publications from 2009 to 2025. A combination of keywords and terms was used to formulate the search queries. The terms and keywords used for the search were categorized into three main groups: BCSCs-related terms: BCSCs biomarkers, chemoradiotherapy resistance, EMT, migration, and invasion. Nanoparticle/delivery-related terms: nanoparticles, nanocarriers, drug delivery, nanomedicine. Metal nanoparticle–related terms: Gold, Copper, Iron Oxide, Silver, Platinum, Zinc, Magnesium, Nickel, and Cobalt. Original research articles, review articles, and book chapters published in peer-reviewed journals were included in the literature.

### Breast cancer stem cells and their markers

**Fig. 1:**
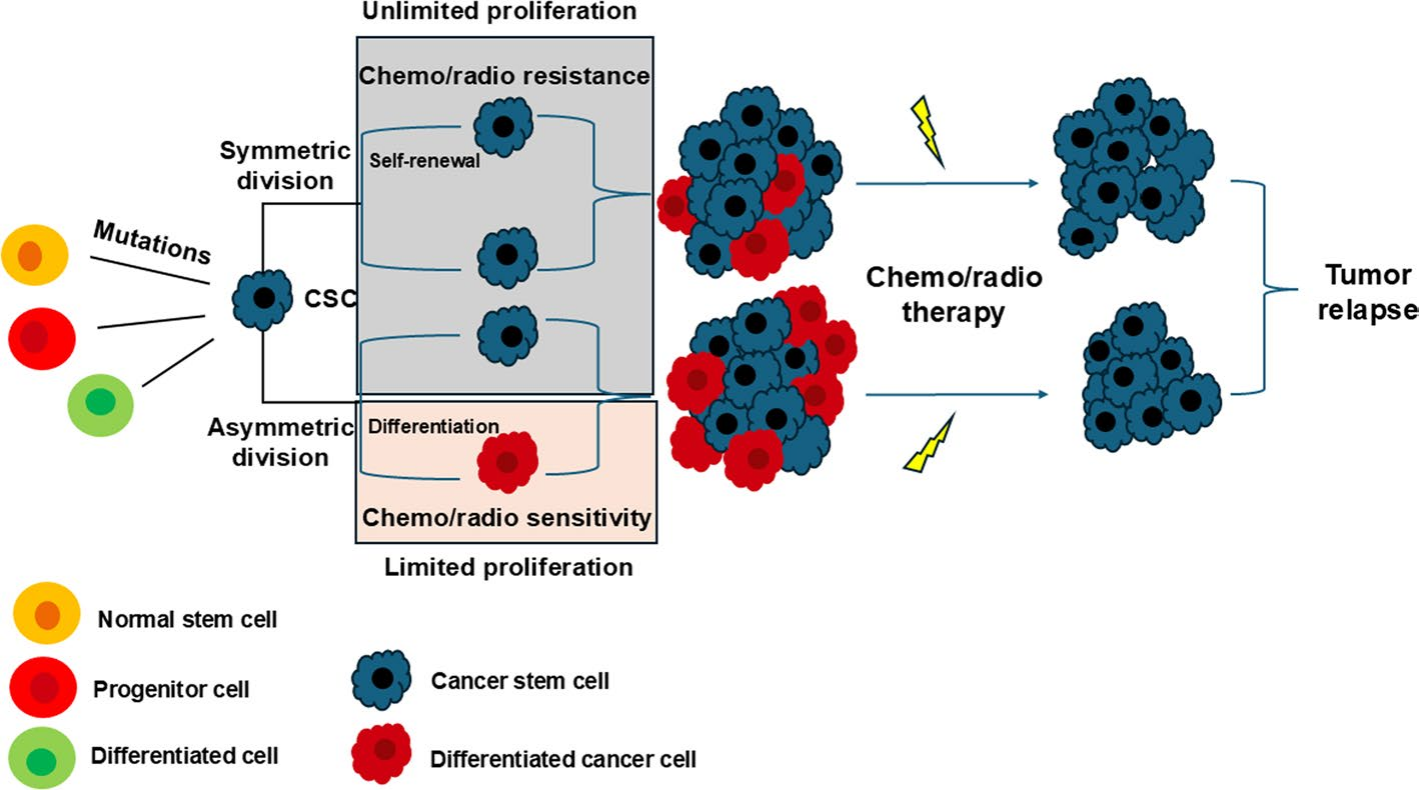
CSCs originated from mutations in normal stem cells, progenitor cells, and differentiated cells. They exhibit symmetric and asymmetric divisions that lead to unlimited and limited proliferation, respectively. CSCs promote tumor relapse due to their potential to survive against chemo/radiotherapy

The presence of heterogeneous cancer cell populations in breast cancer tissues complicates effective treatment. Among these populations, BCSCs represent a distinct subset characterized by stemness-associated properties, including self-renewal, differentiation, metastasis, migration, and resistance to therapy, which contribute to tumor progression and aggressiveness [22, 23]. Dysregulation of signaling pathways such as MAPK, PI3K/Akt/ NFκB, TGF-β, Hedgehog, Notch, Wnt/β-catenin and Hippo pathway as a result of genetic and epigenetic alterations stimulates the transformation of normal stem cells, progenitor cells, or differentiated cells into CSCs [22, 24]. CSCs exhibit symmetrical and asymmetrical division, which promotes unlimited proliferation and drives chemo- and radiotherapy resistance through stemness-associated survival mechanisms (Fig. 1) [25].

Breast tumors are estimated to contain approximately 0.1% to 5% BCSCs whose intrinsic resistance to chemotherapy and radiotherapy is a major factor underlying treatment failure and disease recurrence [22]. Evidence suggests that BCSCs play a critical role in therapeutic resistance, tumor recurrence, and metastasis due to their enhanced DNA repair mechanisms and drug efflux activity via overexpressed ATP-binding cassette transporters (ABCs). Conventional chemotherapeutic approaches predominantly target differentiated cancer cells, often not effective for BCSCs, thereby contributing to treatment failure and disease relapse [10]. Consequently, effective eradication of breast cancer requires therapeutic strategies that specifically target BCSCs.

BCSCs express various specific markers, including CD44+/CD24-, CD326 (EpCAM), ESA, and ALDH activity based on the different types of breast cancer [22]. CD24 and CD44 are considered markers of differentiated breast cancer cells and BCSCs, respectively. CD44, along with interacting with receptor tyrosine kinases (RTKs), interacts with hyaluronic acid (HA) and extracellular matrix proteins such as Osteopontin (OPN) and matrix metalloproteases (MMPs), and regulates signaling pathways involved in cell adhesion, migration, and proliferation of BCSCs [22].

In addition, CD44 triggers activation of signaling pathways such as Rho GTPases, Ras-MAPK, and PI3K/AKT, which are involved in cell adhesion, migration, invasion, and epithelial-mesenchymal transition (EMT) in BCSCs [22, 24]. However, CD44 + alone is insufficient for isolating and detecting BCSCs in all types of breast cancer. ALDH, which plays a crucial role in stem cell differentiation through intracellular aldehyde oxidation, demonstrates a great predictive importance for BCSCs [26].

Epithelial-specific antigen (ESA or CD326) is one of the other crucial markers for identifying BCSCs. This surface protein facilitates cell adhesion, proliferation, migration, and invasion through activating the Wnt signaling pathway [24]. Regulated proteolysis of ESA generates EpCAM intracellular domain (EpICD), which then forms a nuclear complex with Half LIM domains 2 (FHL2) and β-catenin, promoting expression of genes involved in stemness characteristics [22, 27]. Other commonly well-known BCSC markers in various types of breast cancer include CD133, CD166, Lgr5, CD47, and ABCG2 [22, 27].

In addition to the surface markers used to identify BCSCs, recent studies have highlighted the importance of stemness-related transcription factors such as SOX2, OCT4, and NANOG in regulating self-renewal, tumor initiation, proliferation, and therapeutic resistance. Advances in omics technologies, including transcriptomics, proteomics, and single-cell sequencing, have provided deeper insights into BCSC heterogeneity and enabled stronger validation of these developing markers. It has been demonstrated that increased expression of SOX2, OCT4, and NANOG correlates with stem cell-like properties, poor clinical prognosis, and resistance to conventional therapies in breast cancer [28].

Collectively, these unique molecular and surface markers of BCSCs provide an opportunity for selective targeting using nanoparticle-based drug delivery systems. Metal nanoparticles offer advantages such as tunable surface functionalization, enhanced cellular uptake, and controlled drug release, making them promising carriers for the targeted elimination of BCSCs.

### Metal nanoparticles

Nanotechnology has developed as a noticeable area of research due to the specific mechanical, electromagnetic, and optical properties revealed by nanoscale materials [29–33]. Nanoparticles exhibit unique physicochemical properties due to their high surface area-to-volume ratio, which provides a large interface for potential interactions. However, specific binding and interaction with target molecules are primarily mediated by surface functionalization or coating/capping of the nanoparticles. These characteristics make them ideal candidates for drug delivery systems, enabling efficient delivery of therapeutic agents while minimizing side effects [34–36]. Similarity of the size of nanoparticles to the cell organelles allows for targeted drug delivery, improving effectiveness and reducing cytotoxicity associated with conventional chemotherapy. Nanotechnology-driven drug delivery systems offer a promising opportunity for safer and more effective therapeutic interventions [37]. The production of nanostructured metal oxides has recently garnered significant attention due to their distinctive characteristics, including a high surface-to-volume ratio, high surface reactivity, and unconventional electric properties. Metal oxides find extensive application in electronic and photonic devices, medicine, and as catalysts and photocatalysts. They have demonstrated particular utility in numerous scientific domains, such as material sciences, chemistry, electronics, physics, and medicine [38]. MNPs, in particular gold, copper, silver, iron oxide, and zinc oxide, show potential in reducing side effects often observed with traditional treatments of cancer [39, 40]. Despite extensive investigations into metal and metal oxide nanoparticles for biomedical applications, most reported studies remain primarily descriptive and focus on isolated advantages, such as enhanced cytotoxicity or drug-delivery efficiency. However, direct comparison between different metal-based nanoplatforms is often challenging due to variations in experimental conditions, particle size, surface functionalization, and biological models. Moreover, many promising outcomes are derived from in vitro studies, while in vivo validation and long-term toxicity assessments are still limited. These gaps highlight the need for a more critical evaluation of reported findings rather than a purely descriptive overview.

The size, shape, surface, and unique properties of MNPs make them highly reactive and versatile for a variety of applications, including catalysis, electronics, medicine, and environmental remediation [41–44]. These physicochemical characteristics are largely determined by the synthesis approach, and MNPs can be produced via chemical methods (such as chemical reduction and co-precipitation), physical techniques (including laser ablation and evaporation–condensation), and biological or green synthesis routes, allowing precise control over nanoparticle morphology and surface properties [45–48]. The small size of MNPs allows them to catalyze chemical reactions or be used as sensors for identifying specific molecules [49]. MNPs are highly promising for biomedical applications due to their unique magnetic properties, particularly superparamagnetism, which enables their use as contrast agents in MRI. In addition, these properties allow external magnetic field manipulation for targeted drug delivery and image-guided therapeutic applications [50, 51]. Gold and silver nanoparticles exhibit strong localized surface plasmon resonance (LSPR), enabling their use in optical, photoacoustic, and fluorescence imaging, as well as theranostic applications in cancer diagnosis and treatment [52]. This enhanced imaging potential is due to LSPR-mediated absorption and scattering, which improves contrast and sensitivity in multiple imaging modalities [53]. In addition, gold nanoparticles have been shown to support advanced imaging techniques such as two-photon luminescence and dark-field microscopy in cancer theranostics [54]. Plasmonic nanoparticles can also be integrated into multimodal nanoplatforms by combining them with other imaging agents, enabling simultaneous therapeutic intervention and diagnostic imaging, which is highly relevant for personalized cancer management. Cellular uptake of nanoparticles depends on their size, surface charge, and surface functionalization, and typically occurs through endocytic pathways rather than direct membrane penetration. Furthermore, the pharmacokinetic properties of MNPs can be altered by modifying their surface. For instance, coating MNPs with polyethylene glycol (PEG) reduces non-specific uptake by the mononuclear phagocyte system, thereby extending their circulation time within the body [55].

**Fig. 2:**
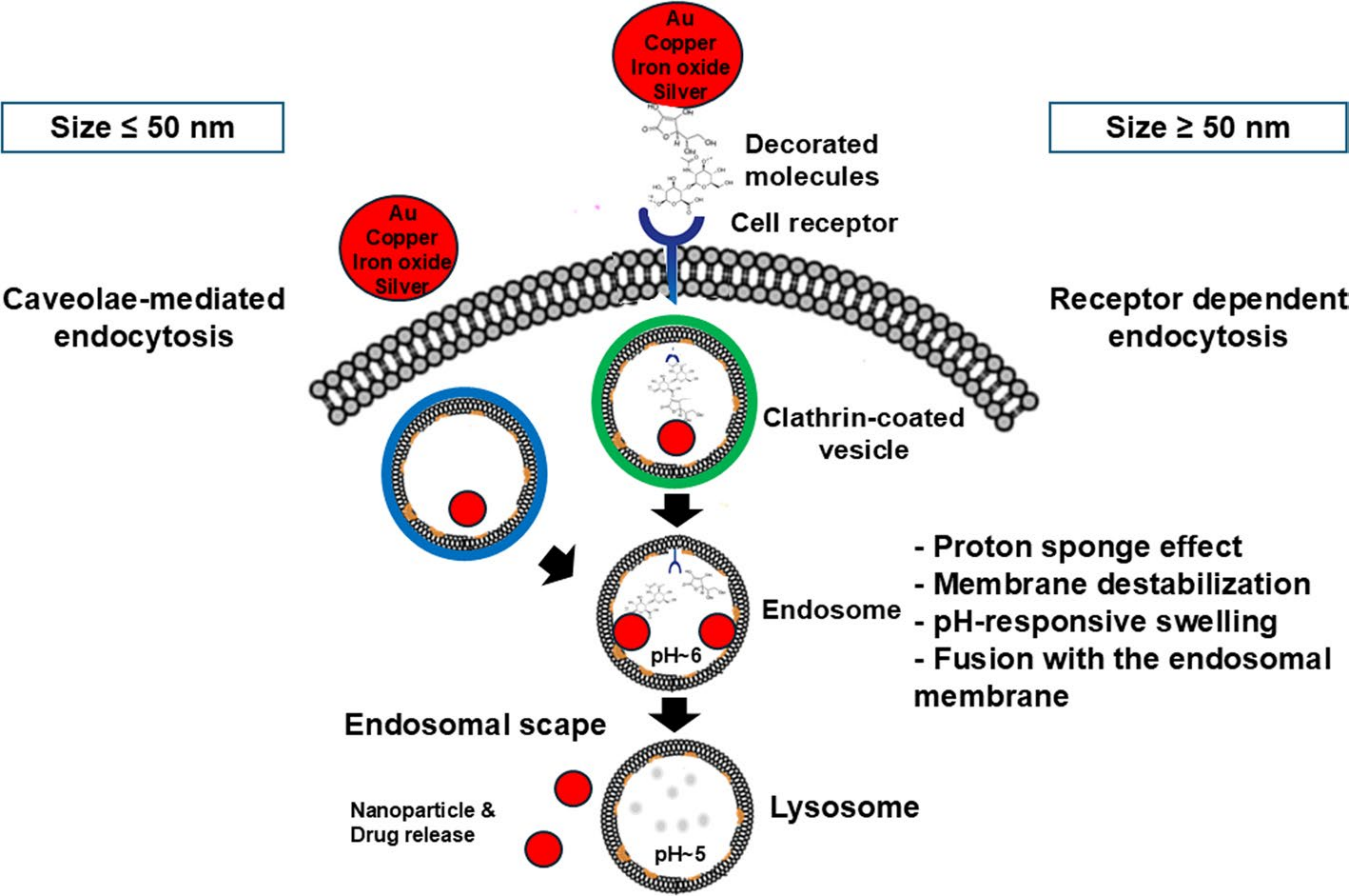
Nanoparticles enter cells via multiple endocytic pathways and are trafficked from endosomes to lysosomes. Engineering nanoparticles for endosomal escape enables cytosolic release of their cargo, enhancing intracellular delivery and therapeutic efficacy

In the medical approach, MNPs are used in targeted drug delivery systems and cancer treatments. Their ability to enhance drug efficiency through targeted delivery, reduce multidrug resistance, and improve therapeutic application [56]. MNP-based drug delivery systems offer several advantages, including increased stability and circulation half-life of nanoparticles, regulated biodistribution, and the ability to target specific molecules in cancer cells. While traditional treatments are common strategies for cancer, they often lack selectivity and result in severe side effects. The specific biochemical and pathological characteristics of tumor tissues compared to healthy tissues provide an opportunity for designing MNPs that specifically target cancer cells [17, 57]. They can be functionalized with specific ligands such as antibodies and peptides that target the specific molecules in cancer cells, improving drug accumulation at the tumor site and reducing cytotoxicity [58]. Mechanistically, metal nanoparticles with targeted coating enter cells mainly through endocytic pathways, including receptor-dependent or clathrin-mediated endocytosis and caveolae-mediated endocytosis, depending on their size, surface charge, and surface ligands. After cell internalization, nanoparticles are enclosed within early endosomes, which then mature into late endosomes characterized by advanced acidification and enzymatic activity. They are eventually trafficked to lysosomes for degradation. To achieve effective intracellular delivery, many nanoparticles are engineered for endosomal escape, using mechanisms such as the proton sponge effect, membrane destabilization, pH-responsive swelling, or fusion with the endosomal membrane. Endosomal escape allows nanoparticles or their cargo to be released into the cytosol before lysosomal degradation, which is critical for enhancing therapeutic efficacy, especially for gene delivery, drug delivery, and radiosensitization applications (Fig. 2) [59]. Metal nanoparticles have been recognized as modulators of epigenetic regulation, raising important considerations for both their therapeutic potential and biological safety [60]. Studies have demonstrated that nanoparticles can influence DNA methylation patterns, histone modifications, and non-coding RNA expression, thereby altering gene expression. These epigenetic effects may arise through nanoparticle-induced oxidative stress, inflammatory signaling, or direct interactions with chromatin-associated proteins. These mechanisms can alter the expression of genes related to cancer, including stemness-related genes. Consequently, understanding nanoparticle-driven epigenetic changes is essential for evaluating long-term health effects, transgenerational risks, and biosafety, as well as for the rational design of nanomedicines with controlled and predictable biological outcomes [61].

MNPs have appeared as a promising approach in cancer drug delivery due to their unique physicochemical properties. They offer a large surface area-to-volume ratio, allowing them to carry significant values of therapeutic agents. MNPs can be designed to release drugs in a controlled manner, either through external stimuli (light or heat) or in response to the tumor microenvironment [17]. Some drugs have decreased water solubility, limiting their bioavailability. MNPs can enhance the solubility of these drugs, improving their delivery and efficiency. They can be engineered to perform multiple functions simultaneously, such as drug delivery, imaging, and hyperthermia treatment [17]. Totally, the unique properties of MNPs, including their large surface area, small size, high permeability, and versatile surface functionality, make them highly attractive for biomedical applications. From a practical perspective, the therapeutic potential of metal-based NPs depends not only on their physicochemical characteristics but also on factors such as selective targeting of cancer cells, biodistribution patterns, clearance mechanisms, and overall clinical feasibility. While certain NPs demonstrate remarkable anti-cancer activity, issues such as non-specific toxicity, bioaccumulation, and lack of standardized evaluation protocols remain significant barriers to clinical application. Rather than providing a purely descriptive summary of previous studies, this review critically evaluates individual metal and metal oxide nanoparticle systems by examining their therapeutic strengths, existing limitations, and unresolved scientific challenges. Each nanoparticle class is discussed independently to enable a clearer comparative assessment of its therapeutic relevance and translational potential in cancer treatment. Metal-based and metal oxide NPs have received growing attention in breast cancer therapy due to their unique physicochemical properties, multifunctionality, and potential for translational prospects. Accordingly, this review focuses on selected nanoparticle platforms, including copper-, iron oxide-, and silver-based systems. These materials have been chosen based on increasing evidence demonstrating their ability to target BCSCs, regulate the tumor microenvironment, and improve therapeutic outcomes through mechanisms such as reactive oxygen species (ROS) generation, oxidative stress, mitochondrial dysfunction, membrane damage, and inflammatory responses, stimulus-responsive drug delivery, and synergistic therapeutic combinations.

Importantly, these nanoparticle systems represent different stages of technological and biological development. Some platforms, such as iron oxide nanoparticles, have progressed toward advanced preclinical evaluation due to their magnetic targeting capability, biocompatibility, and multifunctional therapeutic potential. Other systems, including copper- and silver-based nanoparticles, remain largely at the proof-of-concept or early preclinical stage, offering potent anti-cancer activity but facing challenges related to toxicity control, stability, and targeted delivery. By presenting these nanoparticle classes within a unified framework, this review aims to provide a comparative perspective on their therapeutic promise, limitations, and practical applicability for targeting BCSCs, thereby highlighting key considerations for future translational development in breast cancer treatment.

### Application of gold nanoparticles in drug delivery to BCSCs

Gold nanoparticles (AuNPs) have developed as a versatile tool with a wide range of applications in medicine [62, 63]. Their unique properties, such as their size-dependent optical properties, high surface area-to-volume ratio, and biocompatibility, make them suitable for biomedical applications [64]. AuNPs exhibit high localized surface plasmon resonance (LSPR), which allows them to absorb and scatter light at specific wavelengths. This property can be used for various applications, including imaging. In addition, AuNPs can convert light energy into heat, which can be applied to destroy cancer cells. AuNPs can be functionalized with specific ligands to identify various biological molecules, such as proteins and DNA [62, 63, 65]. Furthermore, previous research has highlighted the efficacy of low-intensity pulsed ultrasound as a potent physical stimulus in amplifying the cellular accumulation of drug-bearing AuNPs via plasma membrane permeabilization [66].

The large surface area of AuNPs enables highly efficient loading of drugs, genes, or other therapeutic agents. AuNPs are considered biocompatible and have been shown to have low cytotoxicity in both in vitro and in vivo experiments, making them suitable for application as a drug delivery system [62, 63, 67]. The surface of AuNPs can be modified with various functional groups, such as thiol, amine, and carboxylic acid groups. This helps the attachment of specific ligands, such as antibodies, peptides, and aptamers, to target specific cells or tissues [68, 69].

#### Gold nanoparticles with photothermal therapy against BCSCs

**Fig. 3:**
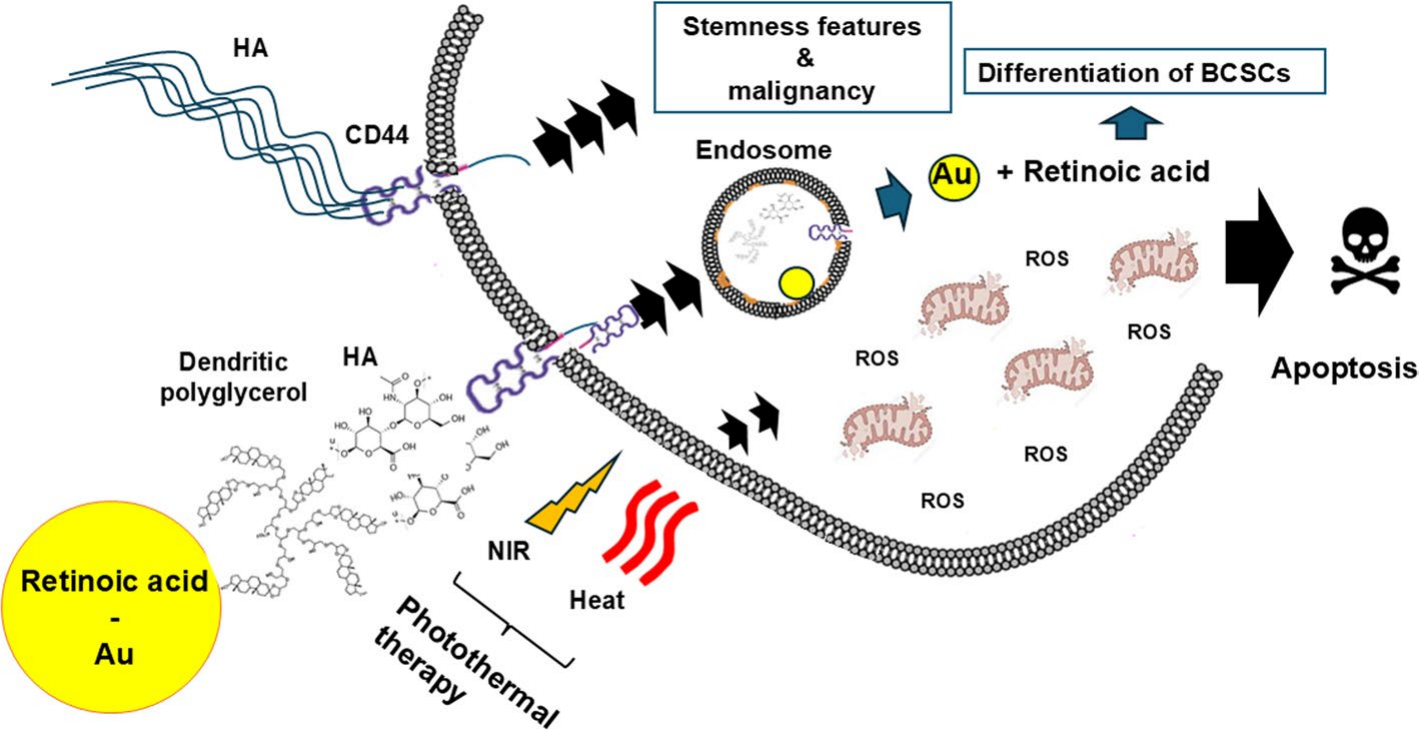
Gold nanoparticles with photothermal therapy against BCSCs. Gold nanoparticles decorated with specific molecules and ligands to target BCSCs. Gold nanostars-dendritic polyglycerol (GNSs-dPG) show specific targeting of CD44 receptor on BCSCs via multiple HA attachment sites on dendritic polyglycerol. Also, Gold nanoparticles can generate heat when exposed to NIR, leading to mitochondrial damage, oxidative stress, and ultimately apoptosis in cancer cells

Recently, researchers have implicated AuNPs as a drug delivery system in the suppression of BCSC [70–73]. The researchers have developed a targeted nanoplatform using retinoic acid-loaded gold nanostars-dendritic polyglycerol (GNSs-dPG) to effectively eliminate BCSCs [70]. Dendritic polyglycerol is a synthetic polymer with a unique, branched structure. It can encapsulate drugs in its structure, protecting them from degradation and enhancing their delivery to target cells. Also, it can create long-circulating nanoparticles in order to release drugs continuously [74–76]. This nanocomposite has exhibited great biocompatibility and targeted capability due to HA decoration on the multiple attachment sites of the bioinert dendritic polyglycerol (dPG), providing an opportunity to target CD44 overexpressed on the surface of BCSCs, specifically. The combination of retinoic acid, which can promote differentiation of BCSCs with photothermal therapy, and targeted delivery has led to the suppression of BCSCs and their stemness features, including self-renewal and tumor growth, stemness gene expression, and mammosphere formation (Fig. 3) [70].

In addition, applying AuNPs decorated with HA as a potential treatment for BCSCs has been developed [71]. The researchers have found that these AuNPs exhibit effective targeting of both differentiated breast cancer cells and BCSCs, with enhanced physicochemical and biological properties compared to traditional anti-cancer drugs. The high efficiency of these nanoparticles has been attributed to the size and ability of the nanoparticles to generate heat when exposed to near-infrared light (NIR, *700–900 nm*), leading to mitochondrial damage, oxidative stress, and ultimately apoptosis in cancer cells (Fig. 3). The biocompatibility of these AuNPs suggests their potential as a novel chemotherapeutic strategy against BCSCs [71]. In addition, the researchers have developed gold nanorods (Au NRs) for targeted therapy of BCSCs [72]. They have found that Au NRs conjugated with polyelectrolytes selectively accumulated within BCSCs, enabling nanoparticles to eliminate BCSCs with photothermal therapy while using NIR irradiation. This treatment significantly reduces the population of BCSCs, as verified by decreased cell subpopulation with ALDH activity, reduced *KLF4* expression, and mammosphere formation ability [72]. Furthermore, Au NRs have been implicated in combination with chemotherapy agents. For instance, AuNRs in combination with salinomycin, a known CSC inhibitor, resulted in synergistic inhibition of CSCs through drug release triggered by NIR and hyperthermia. This novel thermo-chemotherapy platform offers a promising strategy for effectively targeting BCSCs and potentially overcoming the chemoresistance ability of CSCs [72]. On the other hand, combining radiation with gold nanoshells using localized hyperthermia significantly reduces tumor size and suppresses the growth of these types of chemotherapy-resistant cells. It suggests that hyperthermia can enhance the efficiency of radiotherapy in the treatment of BCSC with the radiotherapy resistance ability [77]. Despite the promising efficacy of photothermal therapy, uncontrolled heat generation may induce collateral damage to surrounding healthy tissues, particularly when high laser power densities or prolonged irradiation times are applied. In such cases, heat propagation beyond the tumor margin may cause protein denaturation, membrane disruption, or necrosis in adjacent normal cells. This highlights the critical need for precise temperature control and optimized treatment parameters in clinical applications. Accordingly, strategies such as targeted nanoparticle accumulation, optimized laser parameters, and real-time temperature monitoring have been proposed to mitigate overheating-related risks and improve the safety profile of photothermal-based cancer therapies.

#### Gold nanoparticles in combination with therapeutic agents against BCSCs

**Fig. 4:**
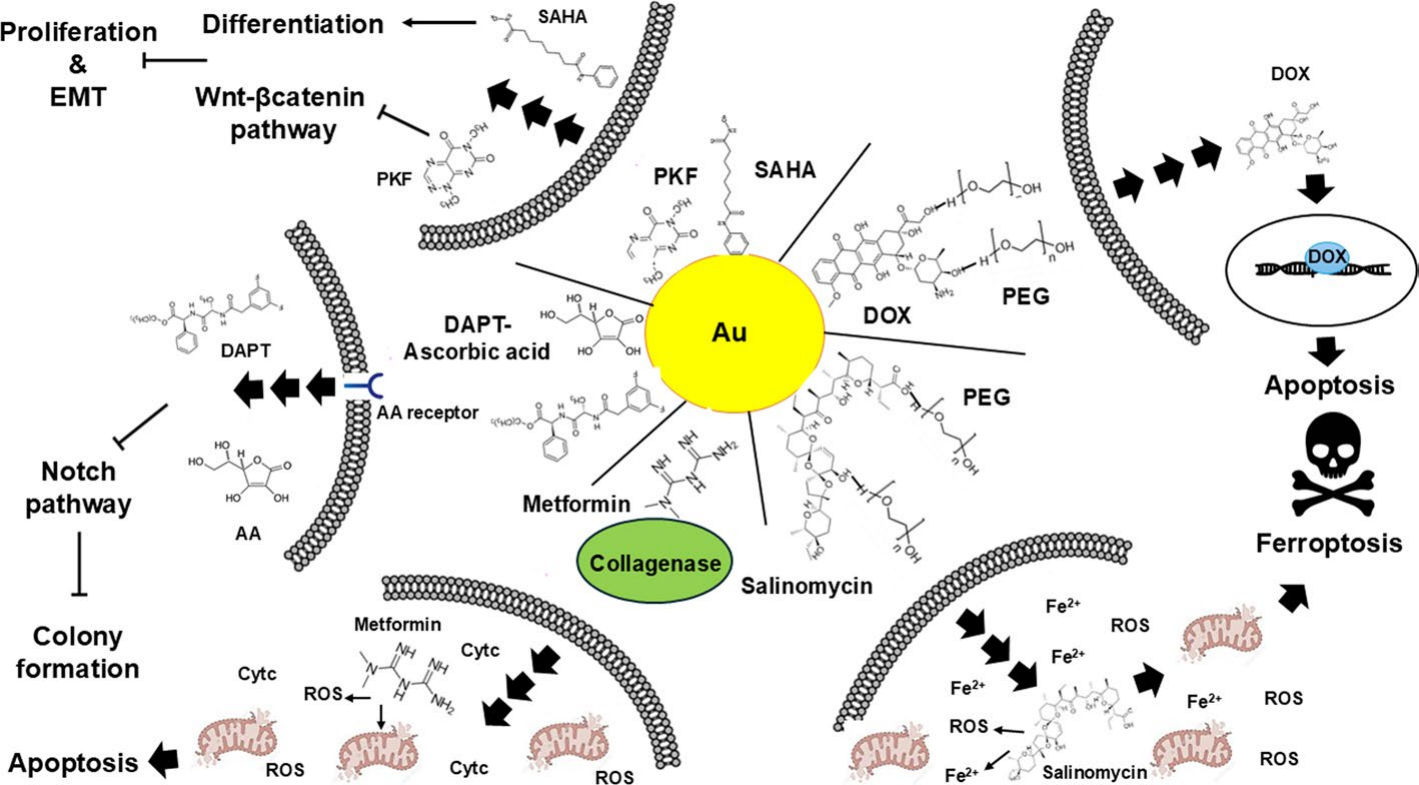
Gold nanoparticles in combination with therapeutic agents against BCSCs. Gold nanoparticles decorated with specific molecules and loaded with drugs for drug delivery to BCSCs. Gold nanoparticles in combination with therapeutic agents against BCSCs

A combination of AuNPs with the chemotherapeutic agents can also be applicable for targeting BCSCs [78–80]. Using doxorubicin (Dox)-loaded spherical AuNPs conjugated with a PEG spacer and an acid-labile hydrazone bond significantly increases Dox delivery to BCSCs, and it ultimately reduces tumor growth, mammosphere formation, and BCSC activity [78]. Dox is an anthracycline that is intercalated between DNA base pairs and blocks replication and translation, resulting in DNA damage in cancer cells (Fig. 4) [81]. This nanostructure inhibits the enrichment of BCSCs, suggesting that this targeted nanoparticle drug delivery holds promise for effective cancer stem cell therapy [78]. Also, it has been demonstrated that BCSCs were highly sensitive to salinomycin- PEG-AuNP treatment. It has been revealed that quasi-spherical salinomycin-PEG-AuNPs induce ferroptosis in BCSCs by accumulating iron and suppressing antioxidant mechanisms. Mechanistically, salinomycin disrupts lysosomal and mitochondrial ion homeostasis, resulting in the release of stored iron as Fe²⁺. Additionally, this process leads to increased mitochondrial ROS, oxidative stress, mitochondrial dysfunction, and lipid oxidation, ultimately resulting in cell death (Fig. 4) [73].

Recently, researchers have implicated the differentiation ability and Wnt-βcatenin pathway activity of BCSCs as potential tools for dual targeting of BCSCs [82]. The researchers propose that a combination of vorinostat or suberoylanilide hydroxamic acid (SAHA), a differentiation agent, and PKF118-310 (PKF), a Wnt-βcatenin pathway inhibitor, can effectively target BCSCs. Their findings demonstrate that spherical AuNPs loaded with both SAHA and PKF exhibit significant cytotoxicity against BCSCs, reducing the stem cell population and suppressing mesenchymal markers, including Snail. This dual-drug delivery system offers a promising strategy for targeting BCSCs and inhibiting tumor growth (Fig. 4) [82].

**Fig. 5:**
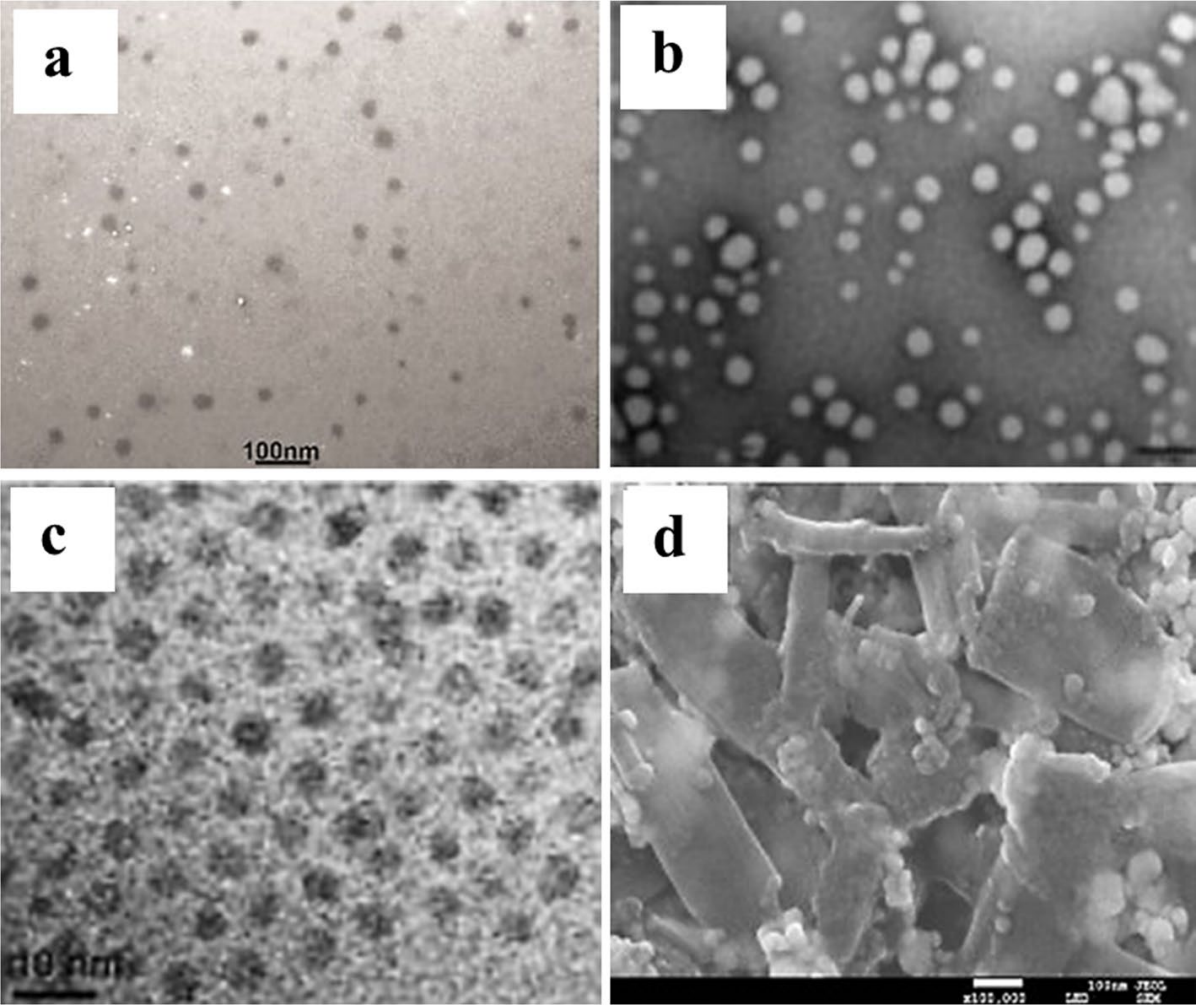
Electron micrographs of nanoparticles. **a** The TEM image of gold nanoparticles [83]; reuse permission was received from Springer Nature, **b** The TEM image of copper nanoparticles [103]; reuse permission was received from MDPI, **C** The TEM image of iron oxide nanoparticles [118]; reuse permission was received from Elsevier, **d **The SEM image of gold/silver nanoparticles [133]; reuse permission was received from ACS publications

The other crucial signaling pathway involved in the proliferation and survival of BCSC is the notch signaling pathway [22]. The researchers have implicated a notch signaling pathway inhibitor in combination with AuNPs in order to target BCSCs [83]. They have functionalized AuNPs with both DAPT, a gamma-secretase inhibitor, and ascorbic acid (AA), which is preferentially taken up by BCSCs due to high expression of AA receptors on the surface of BCSCs. Spherical form of Au-AA-DAPT NPs is observed by electron micrograph (Fig. 5a). It has been confirmed that Au-AA-DAPT NPs significantly inhibit the growth and colony formation of BCSCs (Fig. 4). This suggests that AA enhances DAPT uptake by BCSCs, making this targeted delivery system a promising strategy for effective BCSC elimination and potential cancer treatment [83].

Furthermore, it has been demonstrated that spherical metformin-AuNPs in combination with collagenase significantly exhibit cytotoxic effects on mammospheres compared to metformin alone or metformin-AuNPs [80]. This combination leads to increased apoptosis and decreased collagen within the microenvironment of BCSCs. Mechanistically, metformin increases mitochondrial ROS, leads to mitochondrial membrane depolarization, and promotes cytochrome c release, resulting in activation of caspase-9 and caspase-3. Additionally, it downregulates anti-apoptotic proteins (e.g., Bcl-2) and upregulates pro-apoptotic factors (e.g., Bax), driving apoptosis, particularly in cancer cells and cancer stem cells (Fig. 4) [84]. These findings suggest that targeting the extracellular matrix with collagenase in conjunction with metformin-AuNPs may be a promising strategy for developing breast cancer therapy [80].

Albumin, a natural protein in blood, can be used as a template for gold nanocluster (Au NC) formation. The three-dimensional structure of albumin provides nucleation sites for the growth of Au NCs through interacting with albumin’s specific amino acid sequence, particularly cysteine and histidine residues [85]. It can improve stability, biocompatibility, solubility, and regulated release of Au NCs [86, 87]. Albumin-stabilized Au NCs in combination with Dox and SN38 (Au NCs-DS) release these drugs in response to specific internal stimuli within tumor cells [79]. SN38, as a topoisomerase I inhibitor, prevents cancer cells from cell division and proliferation. It’s synthesized in the body after the administration of irinotecan. Irinotecan is a prodrug, converted into its active form, SN38, once it enters the body [88]. Au NCs-Dox induces significant DNA damage, suggesting a mechanism for their anti-tumor activity in breast cancer. Also, this system effectively reduces the number and the size of mammospheres. These findings indicate that AuNCs-Dox holds promise as a potential treatment strategy for targeting BCSCs [79].

**Fig. 6:**
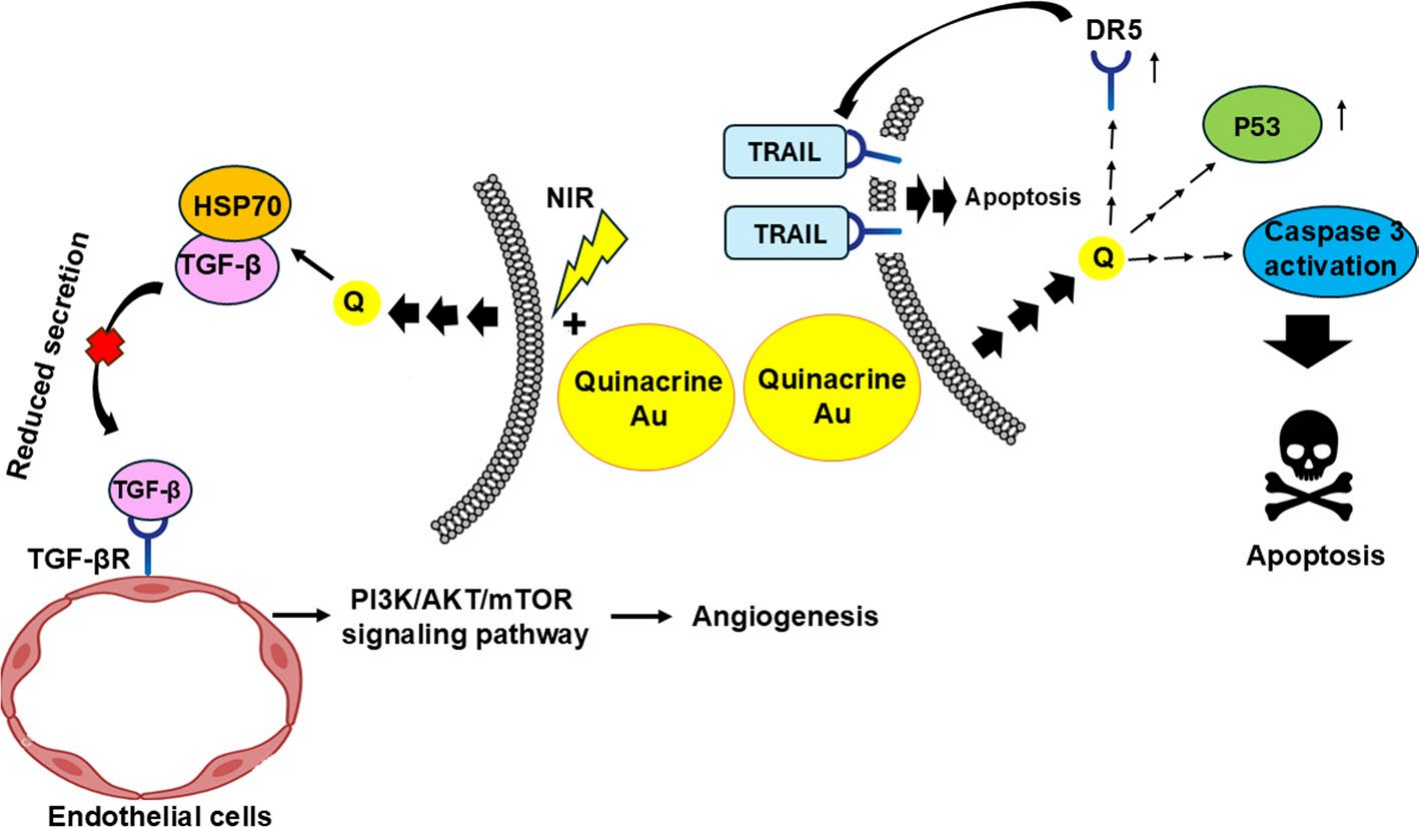
Pro-apoptotic and anti-angiogenic mechanisms of quinacrine–gold hybrid nanoparticles (QAuNPs) in BCSCs. QAuNP treatment enhances apoptosis via upregulation of DR5, activation of p53, and caspase-3. In parallel, QAuNPs + NIR reduces TGF-β secretion. This effect is associated with inhibition of the PI3K/AKT/mTOR signaling pathway and suppression of angiogenesis in endothelial cells

In addition, the anti-angiogenic effects of quinacrine-gold hybrid nanoparticles (QAuNP) in combination with NIR radiation have been verified on BCSCs [89]. Quinacrine, an anti-malarial drug, has shown potential applications in cancer drug delivery: It sensitizes cancer cells to TRIAL-mediated apoptosis through increasing the levels of a specific death receptor 5 (DR5) on the surface of cancer cells [90]. Also, quinacrine induces apoptosis through upregulation of P53 and activation of caspase 3 (Fig. 6) [90, 91]. Researchers have found that QAuNP + NIR treatment deregulates the interaction between HSP-70 and TGF-β proteins within BCSCs. HSP70 acts as a TGF-β inhibitor and plays a suppressive role in the production of VEGF and angiogenesis mediated by TGF-β [92]. This deregulation leads to reduced TGF-β secretion into the tumor environment, subsequently inhibiting angiogenesis through interfering with the PI3K/AKT/mTOR signaling pathway in endothelial cells (Fig. 6). It has been concluded that QAuNP + NIR therapy holds promise as an anti-angiogenic strategy for breast cancer treatment [89].

Recently, the application of herbal medicines in combination with AuNPs has attracted attention for cancer treatment, offering an approach without the side effects and drug resistance associated with chemotherapeutic agents. Due to the low absorption rate, polyherbal formulations (PHFs) have been limited in usage as therapeutic agents in clinical applications. However, conjugation with AuNPs enhances their bioavailability. The researchers have verified the cytotoxicity of AuNP-PHF and AuNP-Dox against paclitaxel-resistant BCSCs. This novel-designed AuNP is also shown to inhibit cell migration and proliferation and promote cell cycle arrest. Also, it induces ferroptosis through downregulation of nuclear receptor coactivator 4 (NCOA4) and upregulation of ferritin and glutathione peroxidase 4 (GPx4), which are recognized as modulators of ferritinophagy. Collectively, it suggests that AuNP-PHF in combination with AuNP-Dox can be applicable as an effective treatment for drug-resistant BCSCs [93].

In addition, a combination of spherical AuNPs with herbal medicines has been investigated in the treatment of breast cancer. It has been reported that two phytocannabinoids, delta-9-tetrahydrocannabinol (THC) and cannabidiol (CBD), have anticancer activities in combination with spherical AuNPs. It can increase bioavailability and uptake of cannabinoids, while decreasing viability of breast cancer cells in 2D and 3D in vitro models and inducing apoptosis in these types of cancer cells [94].

#### Gold nanoparticles in combination with glucose against BCSCs

Currently, MNPs in combination with glucose have been designed to overcome radiotherapy resistance and metastatic potential of BCSCs [95, 96]. Due to the high glucose consumption rate by cancer cells, spherical Glu-AuNPs are designed to target BCSCs, selectively [95]. BCSCs are an important cause of radiotherapy resistance in the treatment of breast cancer patients [22]. The researchers have implicated curcumin in combination with spherical Glu-AuNPs to target BCSCs, directly [96]. It demonstrates that this combination significantly improves the effectiveness of radiotherapy through elevating reactive oxygen species (ROS) levels, reducing expression of stress-related proteins such as HIF-1α and HSP90, and ultimately increasing radiosensitivity and apoptosis in BCSCs, in both normal and under simulated hypoxia conditions. This research suggests that curcumin combined with Glu-GNPs holds promise as a potent radiosensitizer for breast cancer treatment [96]. On the other hand, applying Glu-AuNPs is also appropriate as a targeted therapy for metastasis of BCSCs [95]. Cancer metastasis is a major cause of cancer-related mortality that is facilitated by CSCs [22]. Therefore, targeted therapies specifically targeting molecular pathways of metastasis in CSCs are required. By chemically binding thio-PEG and thio-glucose to AuNPs, the researchers generated functionalized thio-PEG-Glu-AuNPs [95]. Thio-PEGylation, the process of attaching thio-PEG molecules to nanoparticles or drugs, significantly increases their circulation time in the bloodstream due to the low clearance of nanoparticles by the reticuloendothelial system. This prolonged circulation allows for better accumulation of the therapeutic agents in the tumor tissues [5]. In addition, thio-PEG-Glu-AuNPs have demonstrated higher uptake and cytotoxic efficiency in THP-1 cells (a CSC model) compared to MCF-7 breast cancer cells, indicating that the therapeutic effectiveness of Glu-AuNPs is strongly dependent on cancer cell type and metabolic profile. Also, it significantly enhances the efficiency of radiotherapy in these types of cancer cells. Therefore, it could be a promising tool to develop more effective treatments for BCSCs with metastatic and radiotherapy resistance abilities [95].

Moreover, it has been verified that Glu-AuNPs can also be used for targeted delivery of siRNA to BCSCs. The Glu-AuNPs utilize the overexpression of glucose transporter 1 (GLUT1) on the surface of BCSCs for specific recognition and internalization. The researchers have demonstrated enhanced cellular uptake of siRNA payloads by Glu-AuNPs in mammospheres. Applying polo-like kinase 1 (PLK1) siRNA-loaded Glu-AuNPs to tumor-bearing mice resulted in silencing the *PLK1* gene and suppressing tumor growth [97]. PLK1 is a protein kinase, highly expressed in many cancers and associated with aggressive tumor behavior, and plays a critical role in cell division, particularly during mitosis [98].

Totally, AuNPs can be proposed as a potential drug delivery system for targeted therapy of BCSCs through manipulating and decorating NPs with specific molecules that can be applicable for BCSCs alone or in combination with the specific therapeutic agents.

Despite having many advantages, using AuNPs can provide several challenges that may limit the implication of this type of nanoparticle in therapeutic applications [99, 100]. Although AuNPs are generally considered biocompatible, high concentrations or prolonged exposure can lead to cytotoxicity. Also, the production of AuNPs can be expensive, which can limit their widespread use. However, advances in nanotechnology are expected to reduce the cost of AuNP production in the future [99, 100]. Despite these challenges, continued research and development in this area are expected to overcome these challenges and pave the way for the widespread use of AuNPs in therapeutic applications. Despite the promising preclinical outcomes reported for AuNP-based strategies against BCSCs, several important considerations remain. Most studies emphasize in vitro efficacy, mammosphere inhibition, or drug delivery efficiency, while in vivo validation and long-term therapeutic safety are still limited. Differences in particle size, shape, surface functionalization, and experimental models make direct comparisons between different AuNP platforms challenging.

The effectiveness of AuNPs in targeting BCSCs depends not only on their physicochemical properties but also on their selectivity, biodistribution, and clearance. Surface modifications such as conjugation with hyaluronic acid, glucose, or antibodies improve targeting efficiency, yet bioaccumulation, non-specific uptake, and potential dose-dependent toxicity remain critical factors that require careful evaluation. Multifunctional AuNP platforms designed for combinational therapies, although advantageous, introduce additional complexity that can affect reproducibility, scalability, and eventual clinical translation.

Overall, while AuNPs offer a versatile and potent approach for BCSC-targeted therapy, these considerations highlight the need for systematic studies that assess efficacy, safety, and feasibility in more clinically relevant settings. Addressing these challenges will be essential for determining the potential of AuNPs to progress from experimental platforms to practical therapeutic applications.

### Copper-based nanostructures and copper complexes for targeting BCSCs

In addition to copper-based nanoparticles, a growing body of research has explored copper complexes and copper-containing nanostructures as therapeutic agents against BCSCs, owing to their redox activity, ability to generate reactive oxygen species, and capacity to interfere with cancer-related signaling pathways. These copper-based systems exhibit distinct biological behavior compared to noble-metal nanoparticles, largely due to the redox-active nature of copper and its involvement in angiogenesis, oxidative stress regulation, and tumor-associated metabolic pathways [101]. Copper-based nanostructures have been investigated as multifunctional platforms capable of integrating drug delivery with complementary therapeutic strategies, such as photothermal or photodynamic therapy. Importantly, the therapeutic performance of copper-containing systems is strongly influenced by formulation design, oxidation state, and controlled release behavior, which collectively determine their efficacy and safety profiles in cancer treatment [101].

Recently, copper nanoparticles have been used to deliver a copper(II) complex with anti-cancerous activity to BCSCs [102, 103]. The spherical form of the copper (II) complex is observed by electron micrograph (Fig. 5b). Anti-cancer activity of novel copper(II)-phenanthroline complexes in combination with nonsteroidal anti-inflammatory drugs (NSAIDs) such as naproxen, tolfenamic acid, and indomethacin has been investigated [102]. Phenanthroline is an organic compound with the chemical formula C1_2_H_8_N_2_. It is a heterocyclic molecule with a ring structure containing atoms other than carbon. It can bind to metal ions, forming complexes which can have a variety of properties such as stability and some biological activities [104, 105]. These novel nanosystems have shown cytotoxicity against BCSCs better than salinomycin. They induce cell death through ROS generation and cyclooxygenase-2 (COX-2) inhibition in BCSCs, selectively. This research presents the first demonstration of polymeric nanoparticles effectively delivering metal complexes to BCSC [102].

Anti-cancer activity of a novel, stable copper(II) complex containing a bathocuproinedisulfonic acid disodium ligand and two indomethacin moieties has also been verified in BCSCs [106]. Bathocuproinedisulfonic acid disodium is a copper-binding compound being developed for new cancer treatments. This compound may disrupt copper-dependent biological processes in cancer cells. This complex demonstrates potent cytotoxic activity against breast cancer cells and BCSCs rather than salinomycin. It exhibits minimal toxicity towards non-tumorigenic cells. Mechanistically, this complex induces apoptosis in BCSCs by generating ROS. This research highlights the potential of stable copper(II)-NSAID complexes as promising therapeutic agents for targeting BCSCs [106].

The anti-cancer and anti-metastatic effects of a copper(II)-tropolone complex, [Cu(trp)2], have been investigated on human breast cancer cells in both two-dimensional and mammosphere formation [107]. Tropolone is an organic compound with a unique 7-membered ring structure, showing anti-cancer activity through suppressing cell growth and inducing apoptosis. Tropolones can bind to iron, a crucial element for cell growth and survival, and disrupt iron metabolism in cancer cells. Iron chelation can trigger the unfolded protein response (UPR) and cellular stress response in cancer cells [108, 109]. Also, the anti-cancerous activity of tropolones can be mediated by the generation of ROS in cancer cells and dysregulation of expression and activation of proteins involved in apoptosis, such as BCL-2 family proteins [110, 111]. The results have demonstrated that [Cu(trp)2] exhibits significantly higher cytotoxicity against breast cancer cells compared to cisplatin. Furthermore, [Cu(trp)2] inhibits cell migration and metastasis through suppressing metalloproteinase activity and inducing apoptosis. [Cu(trp)2] is significantly more effective than cisplatin against multicellular spheroids, demonstrating its ability to inhibit mammosphere formation and CSC activity [107].

A novel spherical nanoparticle has been designed to combat BCSCs, focusing on encapsulating a discovered copper(II) compound ligated by a bidentate 4,7-diphenyl-1,10-phenanthroline and a tridentate Schiff base ligand within polymeric nanoparticles [112]. These molecules can bind to copper and form a stable complex, as well as enhancing drug delivery efficiency [113]. This encapsulation enhances the agent’s uptake by BCSCs and increases the cytotoxic and immunogenic effects observed with the free drug, as well as generating ROS, endoplasmic reticulum stress, and immunogenic cell death. The nanoparticle formulation results in a significant advancement of the delivery of an immunogenic metal complex to BCSCs using polymeric nanoparticles [112].

Copper also presents some challenges. Copper is a redox-active metal, meaning it can easily gain or lose electrons. This can lead to the formation of ROS, which can damage cellular components, including DNA and proteins. It can accumulate in some organs, such as the liver, and it can show long-term cytotoxicity. Targeted release of copper from nanoparticles is crucial. If copper is released prematurely, it can cause cytotoxicity and reduce the therapeutic efficacy of the drug. Copper can also interact with various biological molecules and pathways, potentially leading to unpredictable side effects. Copper is a relatively expensive metal, which can increase the cost of drug delivery systems. This may limit their accessibility and affordability, especially in resource-limited settings [57, 101]. Despite the encouraging therapeutic performance of copper-based nanoparticles against BCSCs, several important challenges should be carefully considered. Many reported studies focus primarily on cytotoxic efficacy and mammosphere inhibition, while comprehensive in vivo validation and long-term safety assessments remain limited. Variability in nanoparticle formulation, polymer composition, copper complex structure, and experimental models complicates direct comparison between different copper-based nanoplatforms.

The therapeutic effectiveness of copper nanoparticles is strongly influenced by their redox activity, which, while beneficial for ROS-mediated cancer cell killing, may also lead to non-specific oxidative damage in healthy tissues if not precisely controlled. Controlled release mechanisms, selective targeting strategies, and careful optimization of dosage are therefore critical to balance anti-cancer efficacy with systemic toxicity. In addition, the potential accumulation of copper in organs such as the liver raises concerns regarding long-term biocompatibility and clearance.

Overall, although copper-based nanocarriers demonstrate significant promise for targeting BCSCs through diverse mechanisms, including ROS generation, apoptosis induction, and immunogenic cell death, further systematic studies are required to evaluate their safety, reproducibility, and clinical feasibility. Addressing these issues will be essential for advancing copper nanoparticles from experimental drug delivery systems toward practical therapeutic applications in breast cancer treatment.

### Application of iron oxide nanoparticles in drug delivery to BCSCs

Iron oxide refers to a group of chemical compounds composed of iron and oxygen. Iron oxides are versatile materials with diverse biomedical applications. Iron oxides are versatile materials with diverse biomedical applications. In particular, iron oxide nanoparticles (IONPs) have been extensively explored as contrast agents in MRI due to their superparamagnetic properties and ability to enhance image contrast [50].

Iron oxide nanoparticles (IONPs) are used to deliver drugs to specific targets in the body. Some IONPs can generate heat when exposed to magnetic fields, which can be used to destroy cancer cells [114, 115]. Additionally, IONPs can be engineered to release drugs in a controlled manner, either through external stimuli (e.g., magnetic fields, light) or in response to the tumor microenvironment (e.g., pH, temperature) [116]. External magnetic fields can be used to guide IONPs encapsulating drugs directly to the tumor tissue, improving drug concentration and minimizing side effects. Magnetic targeting can significantly increase the accumulation of drugs within the tumor, and IONPs can carry a significant amount of drugs, increasing the therapeutic efficiency. Iron oxide is also a natural substance and is generally considered biocompatible and biodegradable, and minimizes long-term cytotoxicity concerns [116, 117].

**Fig. 7:**
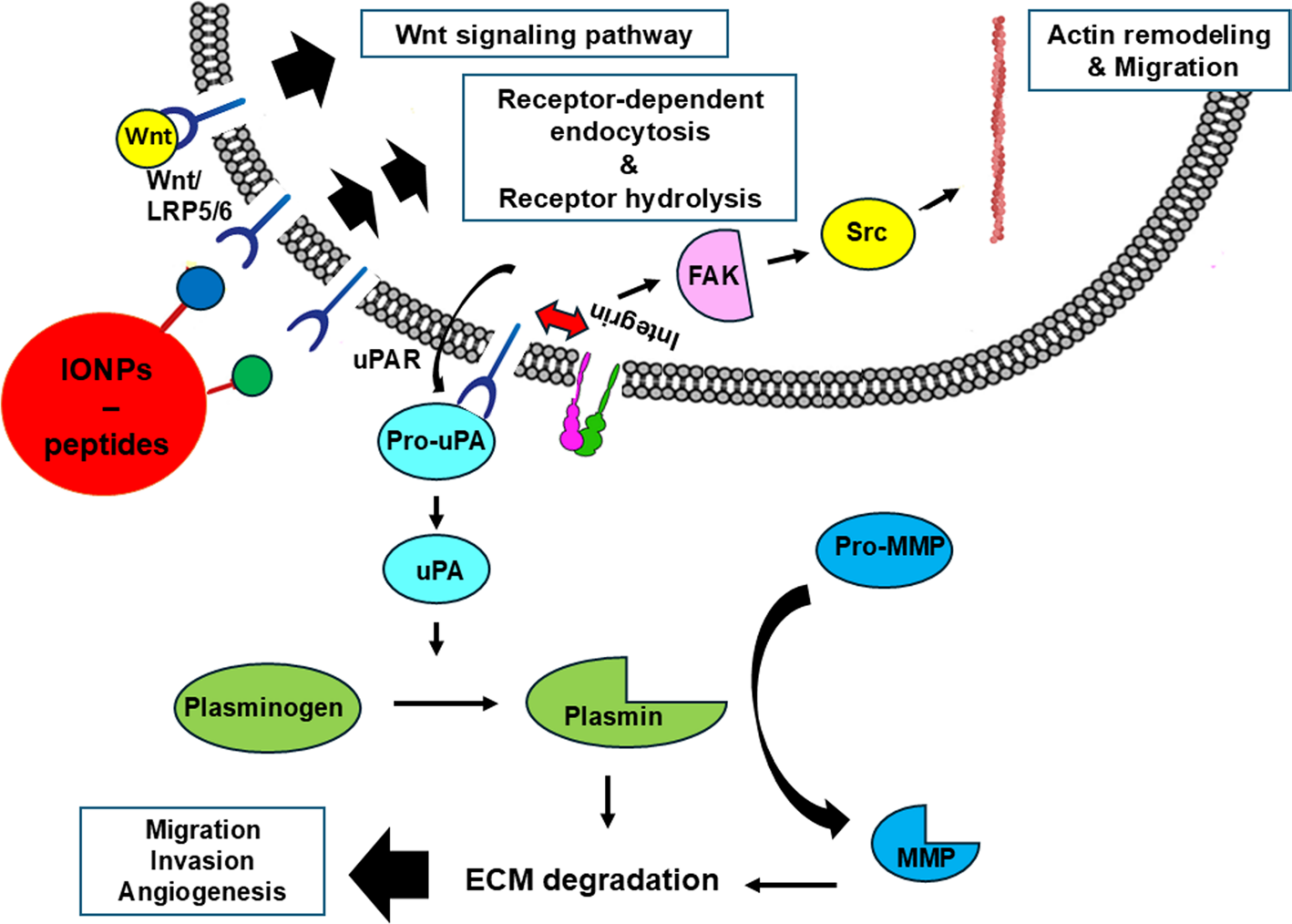
Targeting BCSCs using peptide-conjugated Iron nanoparticles (IONPs). Peptide-functionalized IONPs are designed to selectively target Wnt/LRP5/6 and uPAR receptors overexpressed on BCSCs. uPAR signaling promotes tumor invasion and angiogenesis through uPAR-mediated uPA activation. Activated uPA converts plasminogen into active plasmin, which contributes to ECM degradation and the activation of MMPs. Also, the formation of uPAR–integrin complexes triggers FAK–Src signaling and actin cytoskeleton remodeling, resulting in migration

The researchers have designed ultra-small magnetic spherical IONPs conjugated with peptides, targeting both the Wnt/LRP5/6 receptor and the urokinase plasminogen activator receptor (uPAR) in chemotherapy-resistant BCSCs [118]. The morphology of IONPs is confirmed by electron micrograph (Fig. 5c). uPAR is a protein overexpressed on the surface of cancer cells. It plays a role in a variety of cellular processes, including cell adhesion, migration, invasion, and angiogenesis [119, 120]. Mechanistically, uPAR binds uPA, released by CSCs, and converts plasminogen to plasmin, which directly degrades extracellular matrix (ECM) proteins (fibronectin, laminin, collagen) and activates MMPs, resulting in ECM degradation. In addition, uPAR forms complexes with integrins and activates focal adhesion kinase (FAK)–Src, resulting in actin cytoskeleton remodeling and cell migration (Fig. 7) [121]. The researchers have demonstrated that dual targeting of these receptors by IONPs effectively inhibits cancer cell invasion, suppresses Wnt signaling and stemness characteristics, and leads to significant inhibition of tumor growth. This dual-receptor targeted approach using IONP drug carriers presents a promising therapeutic strategy for overcoming chemoresistance in breast cancer [118].

In addition, IONPs in combination with photothermal therapy have been applied to target BCSCs [122]. It has been demonstrated that photothermal therapy effectively eliminates BCSCs, inhibiting their self-renewal and metastatic spread to the lungs and lymph nodes. Photothermal therapy also induces an immune response against BCSCs [122]. It suggests that photothermal therapy can be used as a potential adjuvant to other traditional treatments. It can eliminate BCSC as well as improve long-term survival in patients with metastatic breast cancer.

Moreover, iron oxide magnetic nanoparticles functionalized with both anti-CD44 antibodies and gemcitabine have been shown to selectively target and bind to CD44-positive cancer cells with high binding affinity. It has been demonstrated that these spherical nanoparticles effectively deliver gemcitabine, leading to the selective killing of CD44+-BCSCs [123]. Gemcitabine is an antimetabolite chemotherapy drug and a nucleoside analog. It is incorporated into the DNA of replicating cancer cells, leading to DNA replication fork collapse [124]. It highlights the potential role of multifunctionalized IONPs as a promising biocompatible platform for targeted cancer therapy.

While IONPs offer significant advantages in drug delivery, they also have some disadvantages [125]. The EPR effect for tumor accumulation can be unpredictable and may not be sufficient for effective drug delivery. While active targeting strategies (e.g., using antibodies and peptides) can improve specificity, developing effective targeting ligands can be challenging and may not always be successful [125]. One of the other negative points of using IONPs is that the depth of magnetic field penetration in tissues is limited, which can restrict the effectiveness of magnetic targeting for deep-seated tumors [126]. Generating a uniform magnetic field within the body can be challenging, especially for irregularly shaped tumors. Although generally considered biocompatible, high doses of IONPs can lead to cytotoxicity, such as oxidative stress and immune responses, in long-term usage [125]. Taken together, these studies indicate that iron oxide nanoparticles represent a multifunctional platform capable of addressing several limitations of conventional cancer therapies, particularly in targeting breast cancer stem cells. Beyond their role as passive drug carriers, the intrinsic magnetic and thermal properties of IONPs enable simultaneous imaging, targeted delivery, and combinational therapeutic modalities. However, the reported outcomes also suggest that therapeutic efficacy strongly depends on nanoparticle size, surface functionalization, and targeting strategy. In particular, dual-receptor targeting and combination therapies appear to outperform single-target approaches, highlighting the importance of rational nanoparticle design rather than material selection alone. These findings underscore the need for standardized evaluation of targeting efficiency, biodistribution, and long-term safety to facilitate the translation of IONP-based systems from preclinical models to clinical applications.

### Application of silver nanoparticles in the drug delivery to BCSCs

Silver nanoparticles have also shown potential in cancer drug delivery due to several key advantages. Silver nanoparticles can increase the solubility of poorly soluble drugs, thereby improving their absorption and distribution within the body [127]. They can be functionalized with ligands (like antibodies or peptides) that specifically bind to receptors on the surface of cancer cells. This targeted delivery improves drug efficacy and minimizes side effects by concentrating the therapeutic agent in the tumor tissue [128, 129]. Silver nanoparticles can be designed to release the drug in a controlled manner, prolonging therapeutic effects and reducing the frequency of administration. Furthermore, silver nanoparticles themselves exhibit intrinsic anti-cancer properties. They can induce cell death through various mechanisms, including oxidative stress, DNA damage, and disruption of cellular processes [130]. In combination with other chemotherapeutic drugs, silver nanoparticles can enhance their anti-cancer activity, potentially providing lower doses of the drugs and reducing side effects [131]. Some silver nanoparticles can absorb light energy and convert it into heat, which can be used to destroy cancer cells through photothermal therapy [132].

A designed hybrid nanoparticle comprising gold/silver decorated with camptothecin, an anti-cancer drug targeting the topoisomerase I enzyme, an enzyme responsible for relieving DNA supercoiling during DNA replication and transcription *has been designed to enhance therapeutic efficacy* [133]. This multidimensional nanosystem combines chemotherapy and thermotherapy, targeting multiple tumor sites and exhibiting diverse cytotoxicity effects on BCSCs. The morphology of gold/silver nanostructures is confirmed by electron micrograph (Fig. 5d). This bimetallic rectangle sheet-like nanosystem effectively reduces the self-renewal capacity of BCSC mammospheres, indicating a decrease in stemness and sensitivity to chemotherapy, and induces apoptosis and necrosis. The anti-cancerous activity of this nanoparticle is attributed to NIR-pH responsive drug release, synergistic thermo-chemotherapy, enhanced cellular uptake of the drug, and silver ion-induced biomembrane damage [133]. This innovative platform offers a potential combinatorial strategy for eliminating BCSCs through the combination of inorganic and organic agents.

Despite applying silver nanoparticles as a drug delivery system, they show some disadvantages [134]. Silver nanoparticles exhibit cytotoxicity and several side effects. They may suppress the immune system and increase the risk of infections. In addition, the behavior of silver nanoparticles within the body can be unpredictable. They may not always reach the target site effectively and can accumulate in undesirable organs. Accumulation of silver nanoparticles in organs such as the liver and kidneys can lead to organ damage. The body may not efficiently clear silver nanoparticles, leading to potential long-term health effects [134]. Collectively, these findings suggest that silver nanoparticles function not only as drug carriers but also as active therapeutic agents with intrinsic anti-cancer activity against breast cancer stem cells. The combination of drug delivery, photothermal effects, and silver ion-mediated cytotoxicity provides a multifaceted approach that may enhance therapeutic outcomes compared to conventional single-modality treatments. However, the therapeutic potential of silver nanoparticles appears to be highly dependent on careful control of dose, surface modification, and hybridization with other materials to balance efficacy and toxicity. In particular, bimetallic and hybrid nanosystems demonstrate improved performance by integrating complementary mechanisms while mitigating some limitations of silver-based platforms. These considerations highlight the importance of rational nanosystem design and comprehensive safety assessment to enable the effective translation of silver nanoparticle-based strategies for targeting BCSCs.

Table 1 presents the metal nanoparticles in combination with specific molecules to target specific markers in BCSCs.

**Table 1:** Metal nanoparticles in combination with specific molecules to target specific molecules in BCSCs

Metal nanoparticles	Synthesis Method	Particle Size	Particle shape	Conjugated/loaded	Direct target	Experimental model	Therapeutic Mechanism	References
Au NP	Seed-mediated growth method (Chemical synthesis of nanostars)	40 nm	Nanostar	Hyaluronic acid/retinoic acid-loaded GNSs-dPG^1^	CD44	MDA-MB-231-derived mammosphere and nude mice	Differentiation of BCSCs/ photothermal therapy	[70]
Au NP	Seed-mediated silver-assisted approach (synthesis of nanostars)	56 nm × 16 nm	Nanorod	Polyelectrolytes/ Salinomycin	N/A	MCF7-derived mammosphere	Decreased ALDH activity, reduced *KLF4* expression and mammosphere formation ability/ Photothermal therapy	[72]
Au NP	Sodium citrate reduction method	∼20 nm	Quasi-spherical	Salinomycin-PEG^2^	N/A	MCF7 isolated BCSCs	ROS-mediated oxidative stress, inducing ferroptosis and suppressing antioxidant mechanisms	[73]
Au NP	Chemical synthesis	30 nm	Spherical	Doxorubicin/PEG and acid-labile hydrazone bond	N/A	MDA-MB-231, BT-474 and MCF-7-derived mammospheres and NOD/SCID mice	Inhibiting mammosphere formation and reducing tumor growth	[78]
Au NP	Seed-mediated growth method	~90 nm	Spherical	SAHA^3^/PKF	Wnt-βcatenin pathway	MCF-7-derived mammospheres	Inhibiting the Wnt-βcatenin signaling pathway, reducing self-renewal and Suppressing EMT	[82]
Au NP	Chemical synthesis	200.4 nm	Spherical	DAPT/ Ascorbic acid AuNP	Gamma-secretase in Notch signaling & Ascorbic acid receptors	HUVEC and MCF-7 isolated BCSCs	Inhibiting the growth and colony formation	[83]
Au NP	Chemical synthesis	∼20 nm	Spherical	Thio-PEG and thio-glucose/AuNPs	GLUT1^4^	THP-1 and MCF7 cell line	Inhibiting metastasis and radiotherapy resistance	[95]
Au NP	Chemical synthesis	∼20 nm	Spherical	Curcumin/Glucose AuNP	GLUT1	MCF-7 and MDA-MB-231-derived mammosphere	Improving the effectiveness of radiotherapy through elevating ROS levels, reducing expression of HIF-1α and HSP90, increasing radiosensitivity and apoptosis	[96]
Au NP	Chemical synthesis	50 nm	N/A	SiRNA PLK1^5^/Glu-AuNPs	GLUT1 & PLK1	MDA-MB-231-derived mammosphere and NOD/SCID mice	Suppressing cell proliferation. Inhibiting tumor growth	[97]
Au NP	Chemical synthesis	35.1 nm and 1.9 nm	N/A	PHF^6^/AuNP and Doxorubicin/AuNP	-	Paclitaxel-resistant MCF7 cell line	Inhibiting cell migration and proliferation, inducing ferroptosis through upregulating ferritin and GPx4 and downregulating NCOA4 and promoting cell cycle arrest	[93]
Albumin-stabilized Au NCs	Chemical synthesis	190 nm	N/A	AuNCs^7/^Dox/SN38	Topoisomerase I	MDA-MB-231 and MCF-derived mammospheres	DNA damage, inhibiting DNA replication and suppressing proliferation	[79]
Au NP	Chemical synthesis	∼49 nm	Spherical	Collagenase/ Metformin/AuNPs	Collagen	JIMT-1-derived mammosphere	Increasing apoptosis, decreasing collagen	[80]
Au NP	Chemical synthesis	∼179 nm	N/A	Quinacrine/ AuNPs	N/A	Patient-derived primary breast cancer stem cells, *in ovo* CAM assay and *BALB/c* mice	Photothermal therapy, inducing apoptosis, inhibiting proliferation and angiogenesis through reducing the expression of TGF-β and deregulating the PI3K/Akt pathway	[89]
Au NP	Chemical synthesis	7–10 nm	Spherical	Phytocannabinoids, THC and CBD	N/A	MDA-MB-231, SK-BR3, and BT-474 in 2D and 3D in vitro models	Decreasing cell viability and inducing apoptosis	[94]
Copper NP	Chemical synthesis	N/A	Spherical	Copper (II)-phenanthroline/ NSAIDs^8^	N/A	CSC-enriched HMLER-shEcad cells	Inducing cell death through ROS generation, inhibiting COX-2^9^	[102]
Copper NP	Chemical synthesis	55.6 nm	Spherical	Copper (II)/bidentate 4,7-diphenyl-1,10-phenanthroline/tridentate Schiff base ligand	N/A	HMLER and HMLER-shEcad-derived mammospheres	Generating ROS, endoplasmic reticulum stress, and immunogenic cell death	[112]
Copper NP	Chemical synthesis	N/A	N/A	Copper (II) complex/bathocuproinedisulfonic acid disodium /two indomethacin moieties	N/A	HMLER and HMLER-shEcad-derived mammospheres	Inducing apoptosis	[106]
Copper NP	Chemical synthesis	N/A	N/A	Copper (II)-tropolone complex	Iron, Metalloproteinase	MDA -MB -231 and MCF-7-derived mammospheres	Unfolded protein response, suppressing cell growth, migration and metastasis, inducing apoptosis	[107]
IONP^10^	Chemical synthesis	23.74 nm	Spherical	IONPs/ Wnt/LRP5/6 receptor/uPAR^11^ ligands	Wnt/LRP5/6 receptor and uPAR	MDA-MB-231 cell line and nude mice	Inhibiting invasion through inhibiting plasmin activation and MMPs and suppressing tumor growth through suppressing Wnt signaling	[118]
IONP	Chemical synthesis	63 nm	Spherical	IONP/ CD44 antibodies/gemcitabine	CD44	MDA-MB231 cell line	Suppressing stemness features	[123]
Au/silver NP	Chemical synthesis	∼337.74 nm	Rectangle sheet-like	Gold/silver NP/camptothecin	Topoisomerase I	Bulk MCF-7 and MDA-MB-231 cell lines	Thermotherapy, NIR-pH responsive drug release, reducing self-renewal, inducing apoptosis and increasing sensitivity to chemotherapy	[133]

^1^ Gold nanostars-dendritic polyglycerol, ^2^ Poly ethylene glycol, ^3^ Suberoylanilide hydroxamic acid, ^4^ Glucose transporter, ^5^ Polo-like kinase, ^6^ Polyherbal formulations, ^7^ Gold nanoclusters, ^8^ Nonsteroidal anti-inflammatory drugs, ^9^ Cyclooxygenase-2, ^10^ Iron oxide nanoparticle, ^11^ Urokinase plasminogen activator receptor

### In vivo studies of metal nanoparticles in breast cancer

In vivo studies have been essential in elucidating the biodistribution, tumor-targeting ability, therapeutic efficacy, and safety profile of metal nanoparticles in breast cancer. Preclinical models, most commonly murine xenograft and orthotopic breast cancer models, provide a physiologically relevant context to evaluate nanoparticle behavior within the complex tumor microenvironment [135]. These studies have demonstrated that metal nanoparticles such as copper, silver, and iron oxide can preferentially accumulate in breast tumors following systemic administration, leading to enhanced anti-tumor efficacy and reduced off-target toxicity [136–138].

A key mechanism underlying tumor accumulation of metal nanoparticles in vivo is the EPR effect, which is particularly noticeable in solid tumors, including breast cancer. Rapid and aberrant tumor angiogenesis results in leaky vasculature characterized by enlarged endothelial gaps and impaired basement membrane integrity. This abnormal vascular structure permits the extravasation of nanoparticles, typically in the size range of 10–200 nm, from the bloodstream into the tumor site. Concurrently, deficient lymphatic drainage within breast tumors limits nanoparticle clearance, promoting prolonged retention and increased local concentration at the tumor site [139].

The EPR effect plays a critical role in facilitating the invasion and penetration of metal nanoparticles into breast cancer tissues, especially in highly vascularized and aggressive tumor subtypes [139]. In vivo imaging and biodistribution studies have shown that nanoparticles use these vascular abnormalities to permeate not only the primary tumor mass but also invasive tumor margins, where cancer cells interact dynamically with stromal components. This accumulation enhances nanoparticle-mediated therapeutic modalities, including photothermal therapy, radiosensitization, drug delivery, and magnetic hyperthermia, by increasing nanoparticle-tumor cell interactions in situ [139].

However, the degree of the EPR effect in breast cancer is highly influenced by tumor size, vascular density, stromal composition, and interstitial fluid pressure. In vivo investigations have therefore emphasized the importance of nanoparticle physicochemical properties such as size, shape, surface charge, and surface functionalization in optimizing tumor penetration and retention [140]. Surface modification strategies, including polymer coating or ligand conjugation, have been shown to prolong systemic circulation and enhance EPR-mediated tumor accumulation while minimizing uptake by the reticuloendothelial system [141].

Overall, in vivo studies highlight the central role of the EPR effect in enabling the passive targeting and invasion of metal nanoparticles in breast cancer. A deeper understanding of tumor-specific vascular and microenvironmental features is essential to improve nanoparticle design and to explain EPR-based nanotherapeutic strategies into clinically effective interventions for breast cancer management.

### Clinical translation of metal-based nanoparticles in breast cancer therapy

While a broad range of metal-based nanoparticles have been investigated preclinically for breast cancer, only a few have advanced into human clinical trials to date, reflecting clinical challenges of these systems. Metal-based nanoparticles can serve as direct therapeutic agents, drug delivery systems, or theranostic enhancers, with clinical approaches. One of the earliest and most widely studied trials involving metal nanoparticles is CYT-6091, a pegylated colloidal gold nanoparticle conjugated with tumor necrosis factor-α (TNF-α). This formulation has been evaluated in phase I clinical trials, involving patients with advanced solid tumors, including breast cancer. CYT-6091 consists of ~ 30 nm gold particles coated with PEG and functionalized with recombinant human TNF-α. Patients have received intravenous infusions with doses ranging from approximately 50 to 600 µg/m² of TNF-α equivalent, administered in repeated cycles (e.g., on day 0 and day 14 of a 28-day cycle) to determine tolerability and the maximum tolerated dose (MTD). Results have demonstrated that doses up to 600 µg/m² have been generally well tolerated with manageable acute toxicities, enabling further pharmacokinetic and pharmacodynamic characterization [142]. Furthermore, iron oxide nanoparticles, particularly superparamagnetic iron oxide nanoparticles (SPIONs), have been evaluated in early clinical studies, mainly as diagnostic and theranostic systems for breast cancer. These nanoparticles, typically composed of Fe₃O₄ or γ-Fe₂O₃ cores with biocompatible coatings, are most commonly administered via intravenous injection. Dosing regimens are generally weight- or surface area-based, with single or repeated administrations depending on formulation and application. Although clinical use has largely focused on imaging rather than therapy, their established systemic safety and pharmacokinetic profiles support further development as drug delivery or image-guided therapeutic systems for breast cancer [143].

Other metallic constructs (e.g., platinum-based nanosystems) are at earlier clinical stages in broad types of cancer but hold potential for future breast cancer applications, especially as drug carriers or therapeutic enhancers [144].

Beyond drug carriers, metallic nanoparticles are also being investigated as radiosensitizers to enhance the efficacy of radiotherapy. While most clinical efforts with hafnium-oxide-based radioenhancers like NBTXR3 are in other solid tumor types, the underlying strategy, local intratumoral injection followed by external beam radiotherapy, illustrates a distinct route of administration for metal-based nanosystems. These agents leverage high-atomic-number metals to amplify radiation dose within tumor tissue (single intratumoral injection before radiotherapy; dose based on tumor volume). Though not exclusive to breast cancer at present, such approaches are under evaluation in broader cancer types. Most nanoparticle systems currently in clinical trials for breast cancer are organic or lipid-based carriers rather than classic metal cores. However, studies have suggested metal nanoparticle systems could offer enhanced targeting and controlled drug release through passive (enhanced permeability and retention) and active targeting strategies, especially when conjugated with targeting ligands or therapeutic payloads [145].

Despite promising preclinical and early-phase clinical data, the clinical translation of metal-based nanoparticles faces substantial regulatory and developmental hurdles. Regulatory agencies such as the U.S. Food and Drug Administration and the European Medicines Agency require rigorous evaluation of nanoparticle pharmacokinetics, biodistribution, long-term toxicity, immunogenicity, and environmental impact. Metal nanoparticles present unique regulatory challenges due to their complex physicochemical properties, batch-to-batch variability, surface modifications, and potential for bioaccumulation. Standardized characterization methods and validated toxicity models remain limited, which further complicates regulatory approval [146].

Additionally, although several nanoparticle platforms have advanced to early-phase clinical trials, there is a notable scarcity of Phase III studies evaluating metal-based nanotherapeutics. This gap reflects challenges, including large-scale Good Manufacturing Practice production, reproducibility, cost-effectiveness, long-term safety concerns, and the difficulty of demonstrating clear superiority over existing therapies. Bridging these translational gaps will require harmonized regulatory guidelines, standardized preclinical testing frameworks, scalable manufacturing strategies, and well-designed multicenter clinical trials to establish safety, efficacy, and cost-benefit profiles necessary for widespread clinical adoption [146].

### Challenges in targeting BCSCs

Despite significant advances in nanotechnology-based drug delivery, several critical challenges remain in effectively targeting BCSCs. BCSCs represent a highly heterogeneous and dynamic subpopulation characterized by phenotypic plasticity, variable marker expression, and the ability to transition between stem-like and non–stem-like conditions. This heterogeneity limits the reliability of single-marker targeting strategies and reduces the efficiency of receptor-specific drug delivery systems [147].

Existing drug delivery systems also encounter substantial limitations, including insufficient tumor penetration, lack of precise cell-specific recognition, off-target accumulation, and nanoparticle-induced toxicity. Passive targeting approaches, such as reliance on the EPR effect, often fail to ensure adequate delivery to deeply embedded BCSC populations. Additionally, physiological barriers within the tumor microenvironment, including dense extracellular matrix, hypoxia, and abnormal vasculature, further restrict nanoparticle distribution and therapeutic efficacy [147].

Therefore, a major unresolved challenge lies in the development of multifunctional and intelligently engineered delivery systems capable of overcoming tumor microenvironment barriers, selectively recognizing heterogeneous BCSC populations, and minimizing systemic toxicity. Addressing these gaps is essential for improving long-term therapeutic outcomes and preventing tumor recurrence and metastasis.

## Conclusion

Breast cancer stem cells (BCSCs) are recognized as a highly malignant subpopulation of tumor cells and a major contributor to therapy resistance and disease recurrence in breast cancer. Consequently, recent advances in targeted and personalized therapeutic strategies have increasingly focused on selectively eliminating BCSCs. In this context, metal-based nanoparticles (MNPs), including gold, copper, iron oxide, and silver nanoparticles, have emerged as promising platforms for targeted drug delivery and combinational therapeutic approaches due to their unique physicochemical properties.

Among these, gold nanoparticles (AuNPs) have been most extensively studied owing to their tunable optical properties, ease of surface functionalization, and biocompatibility, enabling effective drug loading, imaging, and photothermal therapy. Iron oxide nanoparticles (Fe₃O₄ NPs) provide additional advantages through magnetic responsiveness that can be leveraged for targeted delivery and multimodal imaging, while copper-based nanoparticles offer potent redox activity that can be exploited for reactive oxygen species (ROS)-mediated cytotoxicity and photothermal effects. Silver nanoparticles (AgNPs) demonstrate strong antimicrobial and cytotoxic effects that can disrupt cancer cell viability, although this mechanism also raises concerns regarding off-target toxicity in normal tissues.

Functionalization strategies, such as ligand conjugation with antibodies, peptides, or hyaluronic acid, have shown enhanced specificity toward BCSCs by facilitating selective recognition of stemness-associated surface markers. Nonetheless, it is important to underscore that metal nanoparticles do not inherently possess site-specific targeting, and selective delivery relies heavily on appropriate surface engineering.

Despite these promising attributes, significant challenges remain. Dose-dependent cytotoxicity, long-term accumulation, oxidative stress, and unintended biodistribution—particularly in organs of the reticuloendothelial system—are recurring concerns across various MNP platforms. The field also lacks comprehensive mechanistic studies that elucidate how MNPs interact with stemness pathways at the molecular level, and there is a conspicuous absence of late-stage clinical data (e.g., Phase III trials) for MNP-based BCSC-targeted therapies. These limitations hinder translation from preclinical success to clinical application.

Future research should focus on rigorous toxicity profiling, biodistribution studies, scalable and reproducible nanoparticle synthesis, and strategies to enhance both targeting specificity and safety. Integrative approaches combining surface functionalization, controlled drug release, and real-time monitoring may improve therapeutic indices. Continued interdisciplinary efforts aimed at optimizing metal-based nanoparticle design and understanding their biological interactions will be critical for advancing effective and clinically viable strategies targeting breast cancer stem cells.

## Data Availability

No datasets were generated or analysed during the current study.

## Abbreviations

ALDH: Aldehyde dehydrogenase
AA: Ascorbic acid
ABCs: ATP binding cassette transporters
BCSCs: Breast cancer stem cells
CSCs: Cancer stem cells
CBD: Cannabidiol
Cu(trp)2: Copper(II)-tropolone complex
COX-2: Cyclooxygenase-2
DR5: Death receptor5
DPG: Dendritic polyglycerol
EPR: Enhanced permeability and retention
EpICD: EpCAM intracellular domain
EMT: Epithelial-mesenchymal transition
ESA: Epithelial specific antigen
ER: Estrogen receptor
ECM: Extracellular matrix
FHL2: Half LIM domains 2
GLUT1: Glucose transporter 1
GPx4: Glutathione peroxidase 4
AuNCs: Gold nanoclusters
AuNPs: Gold nanoparticles
AuNRs: Gold nanorods
GNSs-dPG: Gold nanostars-dendritic polyglycerol
HER2: Human epidermal growth factor receptor 2
HA: Hyaluronic acid
IONPs: Iron oxide nanoparticles
LSPR: Localized surface plasmon resonance
MRI: Magnetic resonance imaging
MMPs: Matrix metalloproteases
MNPs: Metal nanoparticles
MTD: Maximum tolerated dose
NIR: Near-infrared light
NSAIDs: Nonsteroidal anti-inflammatory drugs
NCOA4: Nuclear receptor coactivator 4
OPN: Osteopontin
PLK1: Polo-like kinase 1
PEG: Polyethylene glycol
PHFs: Polyherbal formulations
PLGA: Poly(lactic-co-glycolic acid
PR: Progesterone receptor
QAuNP: Quinacrine-gold hybrid nanoparticles
ROS: Reactive oxygen species
RTKs: Receptor tyrosine kinases
SAHA: Suberoylanilide hydroxamic acid
SPIONs: Superparamagnetic iron oxide nanoparticles
SPR: Surface plasmon resonance
THC: Delta-9-tetrahydrocannabinol
TNF-α: Tumor necrosis factor-α
UPR: Unfolded protein response
uPAR: Urokinase plasminogen activator receptor
